# Secondary Infection/Microbial Substitution in a Managed Case of Pyogenic Spondylitis

**DOI:** 10.7759/cureus.16432

**Published:** 2021-07-16

**Authors:** Kengo Fujii, Toru Funayama, Sayori Li, Masashi Yamazaki

**Affiliations:** 1 Orthopedic Surgery, Showa General Hospital, Kodaira, JPN; 2 Orthopedic Surgery, Faculty of Medicine, University of Tsukuba, Tsukuba, JPN

**Keywords:** pyogenic spondylitis, percutaneous endoscopic discectomy, posterior percutaneous fixation, anterior debridement fusion, secondary infection

## Abstract

Pyogenic spondylitis is a challenging condition that requires early and accurate diagnosis for appropriate treatment. Most cases can be treated non-surgically or with minimally invasive surgical procedures; however, a combination of anterior debridement/bone grafting and posterior fixation is necessary for severe cases. We encountered a case of lumbar pyogenic spondylitis treated with anterior debridement and autogenous bone grafting after percutaneous endoscopic discectomy drainage (PEDD) with percutaneous pedicle screw (PPS) fixation. The continuous pus oozing from the PEDD drainage tube wound was characteristic in this case, and the pus was considered to be caused by secondary infection/microbial substitution. The discharge immediately stopped and healed after anterior debridement and autogenous bone grafting. *Escherichia coli* was first detected as the causative bacterium, and *Corynebacterium amycolatum* and *Corynebacterium striatum* were detected as the cause of secondary infection/microbial substitution. The possibility of secondary infection/microbial substitution should be considered when the clinical course worsens.

## Introduction

Pyogenic spondylitis (PS) is a challenging condition; immediate and accurate diagnosis followed by appropriate treatment are essential. Treatment options for PS include conservative therapy, rest, and antibiotics for most cases; however, surgical intervention is required for intractable cases, such as progressive neurological deficits, spinal instability, destructive changes, and worsening pain despite adequate antimicrobial therapy [1,2]. Percutaneous drainage is a minimally invasive option [3]. Percutaneous endoscopic discectomy drainage (PEDD) is useful because it is minimally invasive and enables curettage of the infected area under visualization and simple puncture and drainage tube insertion [4-10]. Posterior percutaneous pedicle screw (PPS) fixation is a minimally invasive technique useful for infectious spinal conditions [11-13]. Anterior debridement and autogenous bone grafting are widely performed to achieve both sufficient debridements of the infectious site and stabilization [14-16]. For most cases, PS can be treated with non-surgical procedures or minimally invasive surgical procedures; however, a combination of anterior debridement/bone grafting and posterior fixation is necessary for severe cases.

We report a case of lumbar PS treated with anterior debridement fusion after PEDD and PPS fixation because of the continuous oozing of pus from the postoperative PEDD incision resulting from secondary infection/microbial substitution.

## Case presentation

**Figure 1:**
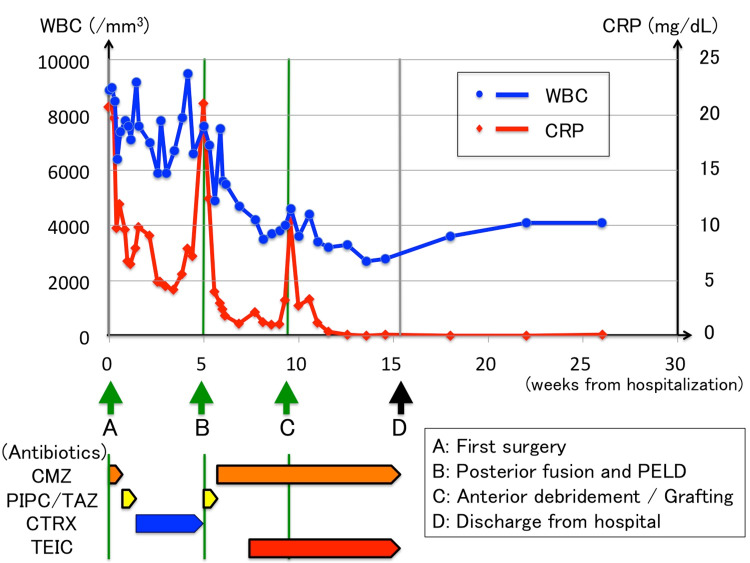
Clinical course and antibiotic administration schematic and graph of the blood test results. The graph shows the changes in the laboratory data, including the white blood cells (WBC) and C-reactive proteins (CRP). Bars and arrows below the graph show the time points of antibiotic administration: (A) first surgery; (B) posterior fusion and percutaneous endoscopic lumbar discectomy/debridement (PELD); (C) anterior debridement/grafting; and (D) discharge from hospital. CMZ, cefmetazole sodium; TAZ/PIPC, tazobactam/piperacillin; CTRX, ceftriaxone sodium; TEIC, teicoplanin

**Figure 2:**
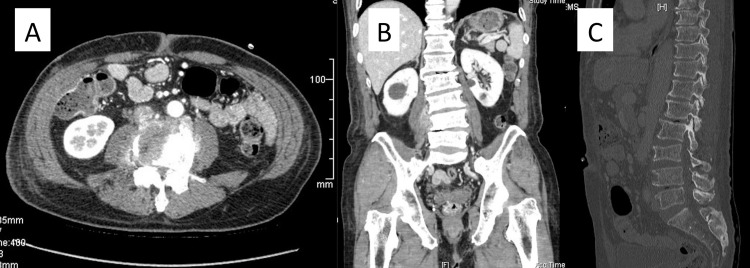
Enhanced computed tomography scan slices. (A) Axial slice shows an enhanced lesion around the L4 vertebral body and right psoas muscle, suggesting the focus of infection. (B) Coronal slice shows an enhanced lesion in the right psoas muscle, suggesting psoas abscess. (C) Sagittal bone quality slice shows no destructive changes in the L3/4 endplates and vertebral bodies.

**Figure 3:**
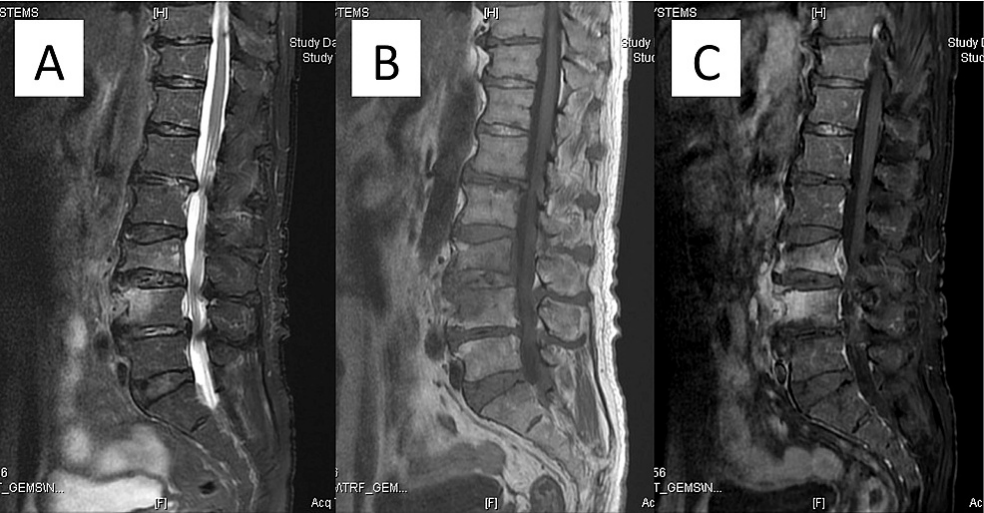
Gadolinium-diethylenetriamine pentaacetic acid (Gd-DTPA)-enhanced lumbar images obtained with magnetic resonance imaging. (A) Sagittal T2-weighted fat-suppressed image shows high-intensity changes in the L3 and L4 vertebral bodies. (B) Sagittal T1-weighted fat-suppressed image shows low-intensity changes in the L3 and L4 vertebral bodies. (C) Sagittal Gd-DTPA-enhanced T1-weighted image shows enhancement in the L3 and L4 vertebral bodies, anterior paravertebral soft tissue, and epidural abscess at the L3 vertebra level.

**Figure 4:**
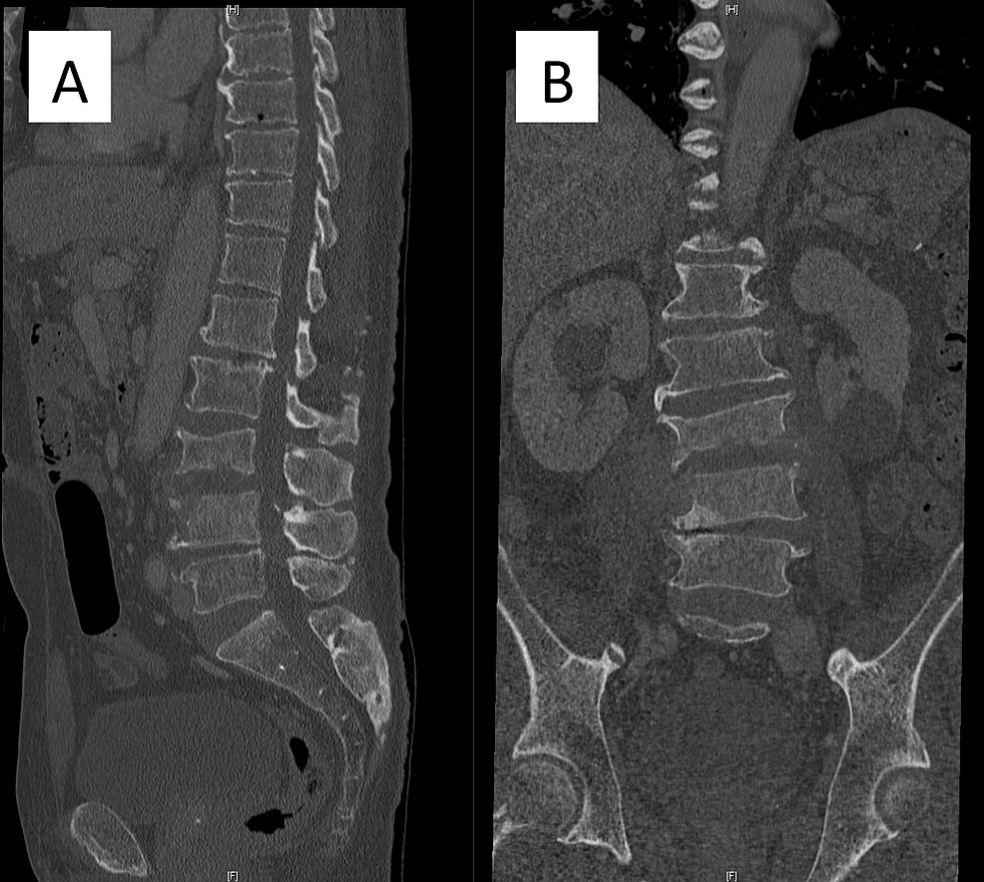
Secondary computed tomography scan slices after four weeks of antibiotics administration. (A) Sagittal slice. (B) Coronal slice. Progressive destructive changes of the L3 and L4 endplates and vertebral body were observed.

**Figure 5:**
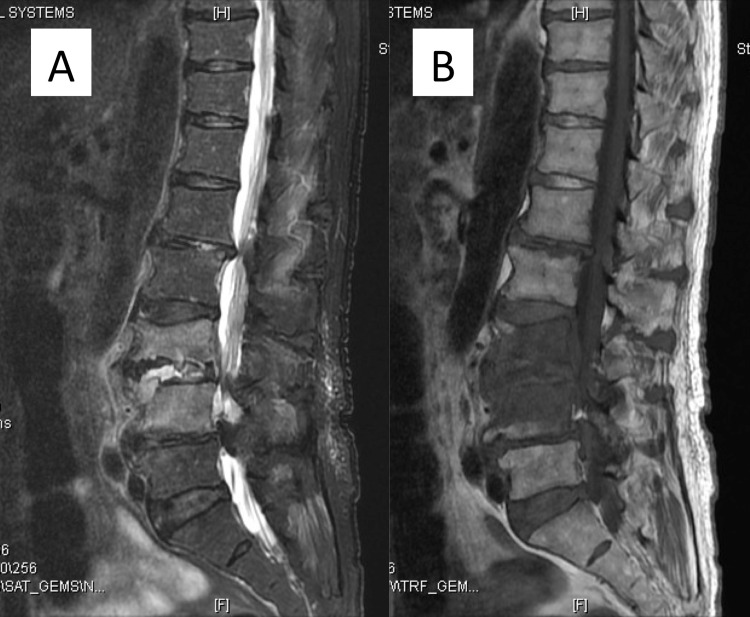
Secondary images obtained with magnetic resonance imaging. (A) Sagittal T2-weighted fat-suppressed image. (B) Sagittal T1-weighted image. Fluid collection in the intervertebral disc space and diffuse signal changes in the L3 and L4 vertebral bodies with destructive changes were observed.

A 78-year-old man experienced fever and back pain five weeks before his first spinal surgery. He did not have any history of diabetes, use of steroids, or any other immunosuppressive conditions instead of gastric cancer. He visited a local clinic two days after onset and was diagnosed with choledocholithiasis cholangitis. He was transferred to our institute the next day for gastrointestinal endoscopic treatment; however, this treatment was unsuccessful because of his previous surgery for gastric cancer. He underwent emergency open cholecystectomy and lithotripsy for common bile duct stones on the same day. After abdominal surgery, fever and back pain continued with high C-reactive protein levels (range: 4.1-20.8 mg/dL), despite intravenous administration of antibiotics (Figure 1). First, tazobactam/piperacillin (TAZ/PIPC) was chosen as an empiric broad antibiotic. It was de-escalated to ceftriaxone (CTRX) after *Escherichia coli* (*E. coli*) was detected in the results of two consecutive blood culture tests (Figure 1). Multiple penicillin and cephem derivatives were sensitive to *E. coli*. Enhanced computed tomography (CT) images to determine the source of inflammation at 11 days after admission revealed highly enhanced signal changes in the L3/4 intervertebral disc and a right psoas muscle abscess connected to the L3/4 disc space (Figure 2). Lumbar magnetic resonance imaging (MRI) showed low-signal changes in the caudal L3 and cranial L4 vertebral bodies on T1-weighted images, high-signal changes on T2 fat-suppressed images, and enhanced gadolinium-diethylenetriamine pentaacetic acid (Gd-DTPA) on T1-weighted images (Figure 3). We chose conservative therapy and continued intravenous CTRX administration (Figure 1). Four weeks after admission, his back pain did not improve and C-reactive protein levels began to increase. Lumbar MRI and CT scans showed progressive destructive changes in the L3 and L4 vertebral bodies (Figures 4, 5).

**Figure 6:**
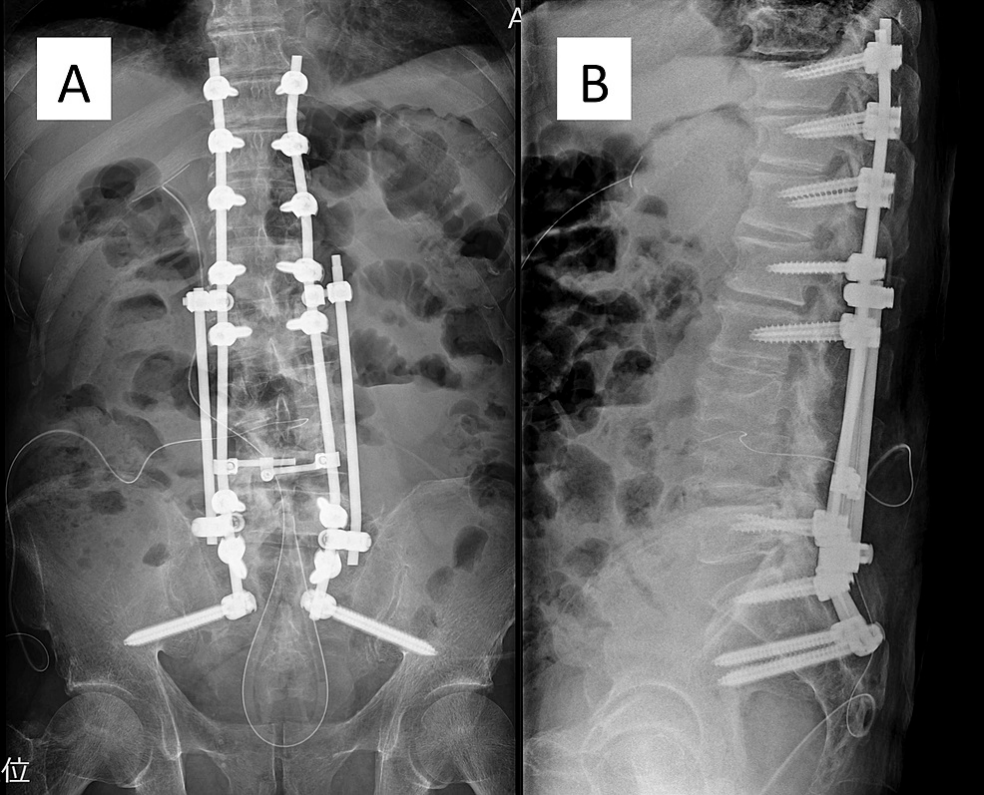
Postoperative X-ray images of posterior percutaneous screw fixation from T10 to the pelvis and percutaneous endoscopic discectomy/debridement to L3/4. (A) Anteroposterior X-ray image. (B) Lateral X-ray image.

We simultaneously performed PPS fixation from T10 to the pelvis and PEDD to L3/4 at 34 days after admission. Bilateral PPS for T10 to L2 and L5, and S1 and bilateral S2-alar-iliac screws were inserted and percutaneously connected to the rod. Two other rods were fixed between the L2 to L5 level with lateral connectors to achieve a four-rod construct (Figure 6). The rod was bent in situ using an intraoperative spinal rod bending system (Bendini; NuVasive Inc., San Diego, CA). PEDD was performed via another incision. In the prone position, percutaneous endoscopic observation, debridement with forceps and a bipolar coagulator, and irrigation were performed for the L3/4 intervertebral disc. Instead of the normal nucleus pulposus, infectious muddy fluid and some floating tissue were observed and collected. A Blake silicon drainage tube (10-Fr, Blake drains and J-VAC Suction Reservoirs; Ethicon Inc., NJ) was placed in the disc space and fixed with a suture. 

*E. coli* was detected again during a culture analysis of the intraoperatively obtained samples. The antibiotic regimen was changed to cefazolin (CEZ) 40 days after admission (Figure 1). 

**Figure 7:**
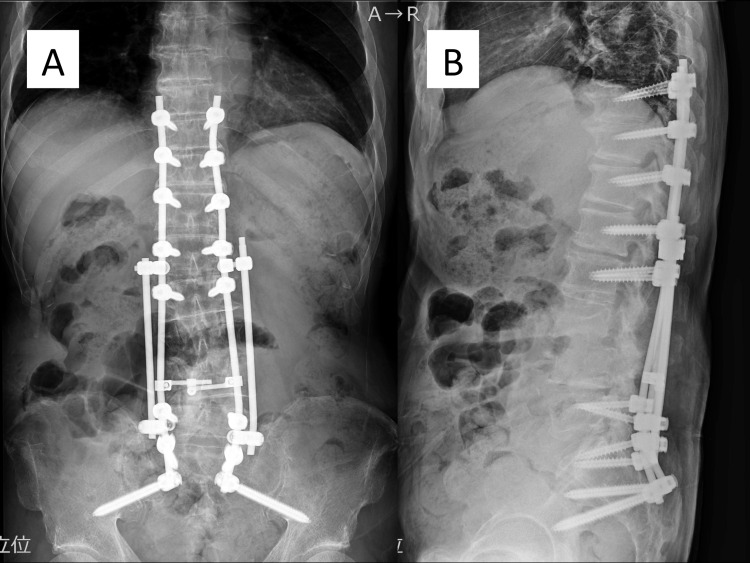
Postoperative X-ray of anterior debridement and autogenous bone grafting to L3/4. (A) Anteroposterior X-ray image. (B) Lateral X-ray image.

**Figure 8:**
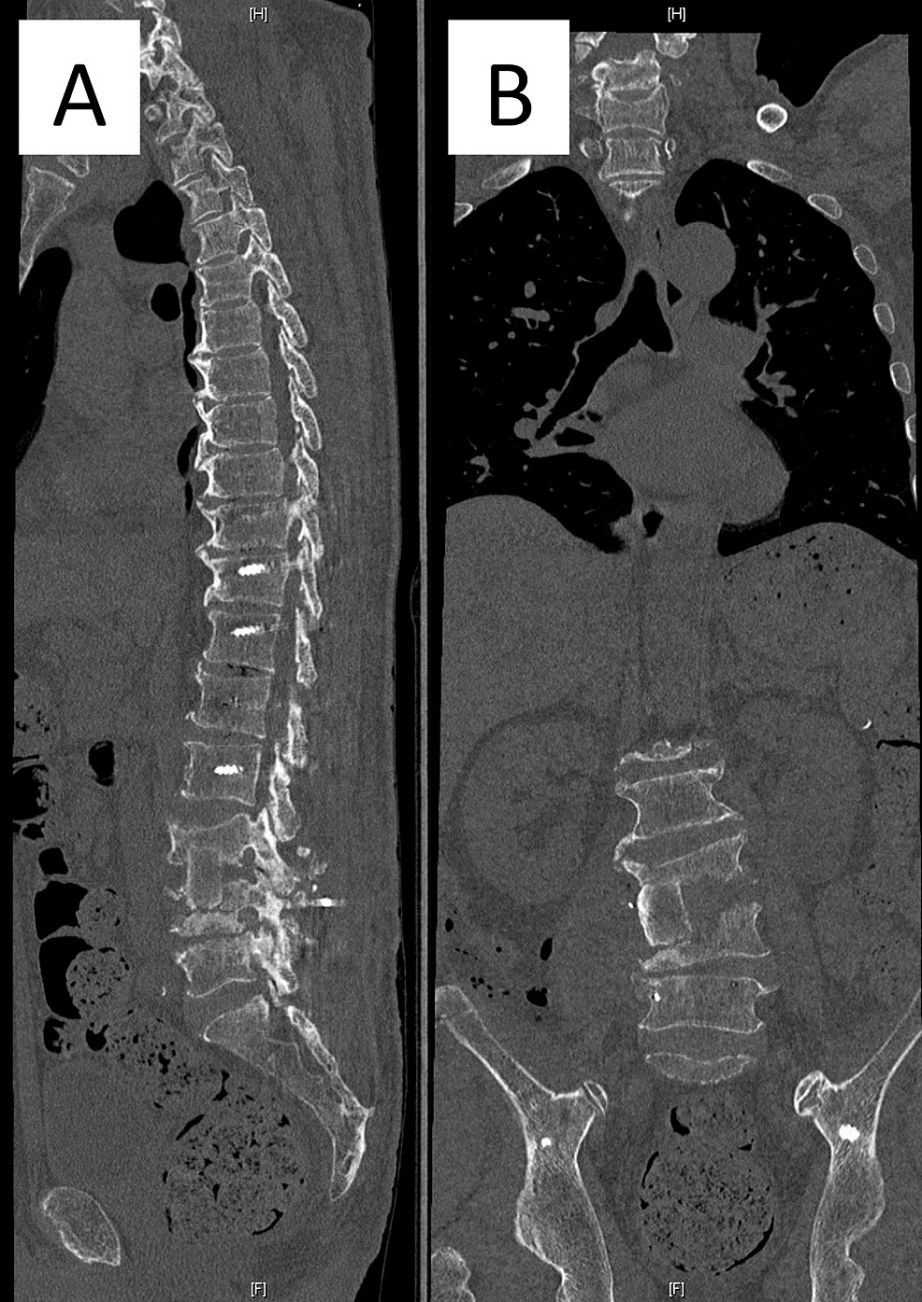
Postoperative computed tomography (CT) images of anterior debridement and autogenous bone grafting to L3/4. (A) Sagittal CT image. (B) Coronal CT image.

**Figure 9:**
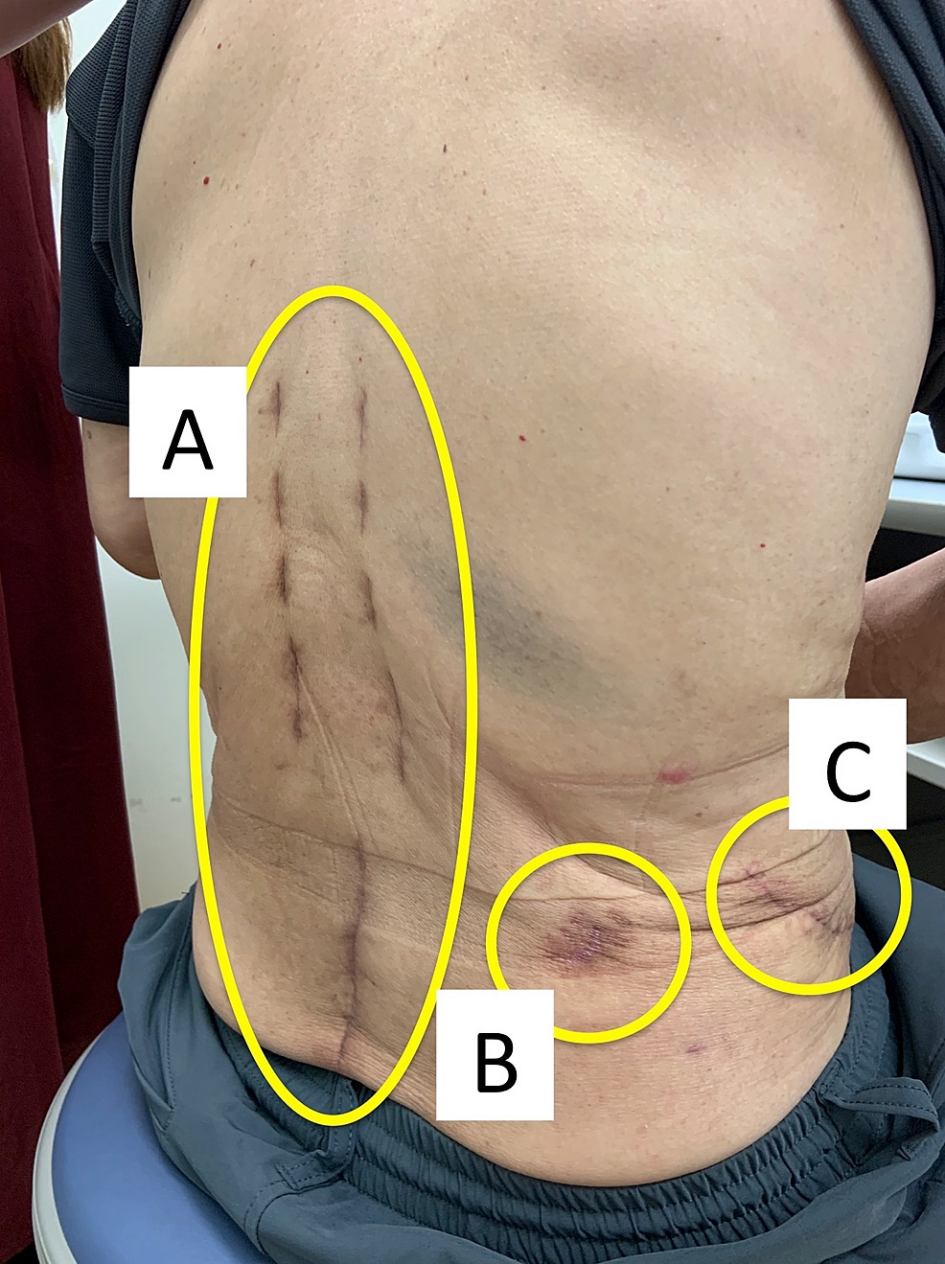
Postoperative scars (A) Scars after posterior percutaneous pedicle screw fixation. (B) Scar after percutaneous endoscopic discectomy/debridement. (C) Scar after anterior debridement and autogenous bone grafting.

His back pain improved immediately and the drainage tube was removed 47 days after admission; however, oozing from the drainage tube wound continued. The fluid was initially serous and gradually changed to purulent fluid. Local debridement with local anesthesia was performed 55 days after admission; *Corynebacterium amycolatum* and *Corynebacterium striatum* were detected during a pus culture analysis. Secondary infection with multidrug-resistant bacteria was diagnosed. Teicoplanin (TEIC) administration was started immediately and CEZ administration was continued (Figure 1). We performed anterior debridement and autogenous bone grafting to L3/4 at 64 days after admission using the retroperitoneal approach because the focus of the secondary infection was considered to be located in the L3/4 disc space and the vertebral body of L3 and L4 (Figures 7, 8). At that point, PEDD from right side had already been performed and failed, and the posterior fusion was already performed, and we could not detect the undoubted reason for the secondary infection or microbial substitution. Therefore, we considered that another surgical option of re-PEDD from the other side might fail to control the infection. An autogenous bone graft was harvested from his right iliac crest. Debridement and suturing of the fistula were also performed. TEIC and CEZ were continued for six weeks after anterior debridement surgery. The CRP level turned negative at 14 weeks after admission. At the follow-up at 12 months after final surgery, the infection did not recur, CRP level stays negative, no spinal instrument failure/loosening and bone union around the autogenous bone graft were observed by CT scans, and the patient does not complain of any symptoms (Figures 1, 9). 

Written informed consent was obtained from the patient for publication of this case report. The study was performed in accordance with the Declaration of Helsinki and within the appropriate ethical framework.

## Discussion

Our patient had a PS treated with PEDD, PPS fusion, anterior debridement, and autogenous bone grafting with characteristic oozing of pus. Multiple surgical procedures, rest, and antibiotics were required. The treatment strategy for PS comprises: identifying the pathogen [2,16]; appropriate antibiotic administration; posterior fusion to achieve local stability; and debridement, including needle puncture, percutaneous endoscopic lumbar discectomy/debridement (PEDD), and anterior debridement fusion.

To identify pathogens, which is the first important step, a blood culture analysis and biopsy of a sample obtained through PED or CT-guided biopsy before starting antibiotic administration are mandatory [2,16]. In this case, *E. coli* was detected during the blood culture analysis. Therefore, it was considered reasonable that PS was hematogenously induced/transferred by choledocholithiasis cholangitis. Intravenous antibiotic administration for six weeks is recommended [2,17]. However, surgical indications and standards for PS are still unclear. Options include posterior fusion, PPS fusion, anterior debridement/fusion, and PEDD. 

When conservative treatment is ineffective and surgical procedures are inevitable, we first consider PEDD and/or PPS fusion; we secondarily consider anterior debridement and autogenous bone grafting. First of all, there is still no consensus on the optimal surgical strategy for PS [2]. Recently, the effectiveness of PPS fusion without anterior debridement is reported [11,12,18,19]; the effectiveness of intradiscal drainage with PEDD has been previously reported [2,4,5,7-10,14,15,20]. There is a recent article reported successful case series treated by one-stage posterior fixation with anterior debridement from posterior approach [21]. When infection focus is limited in anterior and middle column, we usually avoid the anterior interbody/intradiscal procedure via posterior approach because we worry about the iatrogenic infection progress to the posterior area. As a result, we had to perform every aforementioned surgical option because of the poor clinical course. 

*E. coli* was the initial cause of PS in this case. A literature review indicated that PS caused by Gram-negative rod bacteria has good sensitivity to antibiotics and responds well to conservative treatment [22-26]. In this case, secondary infection with *C. amycolatum* and *C. striatum* caused an active infectious response in the L3/4 disc space and positive pressure from the focus of infection resulted in continuous oozing of pus through a tunnel formed in muscle and subcutaneous tissue in the portal route used for PEDD. In this case, retrograde infection via drain tube could be a potential cause of the secondary infection. Secondary infection/microbial substitution in PS should be considered when the clinical course worsens, although this condition is considered uncommon, and the cause and incidence are unknown.

## Conclusions

We reported a case of lumbar pyogenic spondylitis treated with anterior debridement fusion after percutaneous endoscopic discectomy drainage with percutaneous pedicle screw fixation because of the continuous oozing of pus from the postoperative PEDD incision caused by secondary infection/microbial substitution. The possibility of secondary infection/microbial substitution in pyogenic spondylitis should be considered when the clinical course worsens.
